# Photochemotherapy Induces Interferon Type III Expression via STING Pathway

**DOI:** 10.3390/cells9112452

**Published:** 2020-11-10

**Authors:** Edyta Biskup, Brian Daniel Larsen, Leonor Rib, Lasse Folkersen, Omid Niazi, Maria R. Kamstrup, Claus Storgaard Sørensen

**Affiliations:** 1 Department of Dermatology, Bispebjerg Hospital, DK-1014 Copenhagen, Denmark; omidniazi@yahoo.com (O.N.); maria.roerbaek.kamstrup.02@regionh.dk (M.R.K.); 2 Biotech Research and Innovation Centre, University of Copenhagen, DK-1014 Copenhagen, Denmark; brian.larsen@bric.ku.dk (B.D.L.); leonor.rib@bric.ku.dk (L.R.); 3 National Genome Center Denmark, Department of Bioinformatics, DK-1014 Copenhagen, Denmark; lwf@ngc.dk

**Keywords:** DNA damage, STING, PUVA, CTCL, IFNL1

## Abstract

DNA-damaging cancer therapies induce interferon expression and stimulate the immune system, promoting therapy responses. The immune-activating STING (Stimulator of Interferon Genes) pathway is induced when DNA or double-stranded RNA (dsRNA) is detected in the cell cytoplasm, which can be caused by viral infection or by DNA damage following chemo- or radiotherapy. Here, we investigated the responses of cutaneous T-cell lymphoma (CTCL) cells to the clinically applied DNA crosslinking photochemotherapy (combination of 8–methoxypsoralen and UVA light; 8–MOP + UVA). We showed that this treatment evokes interferon expression and that the type III interferon IFNL1 is the major cytokine induced. IFNL1 upregulation is dependent on STING and on the cytoplasmic DNA sensor cyclic GMP-AMP synthase (cGAS). Furthermore, 8–MOP + UVA treatment induced the expression of genes in pathways involved in response to the tumor necrosis factor, innate immune system and acute inflammatory response. Notably, a subset of these genes was under control of the STING–IFNL1 pathway. In conclusion, our data connected DNA damage with immune system activation via the STING pathway and contributed to a better understanding of the effectiveness of photochemotherapy.

## 1. Introduction

Therapeutic modalities like chemo- and radiotherapy cause DNA damage and are particularly toxic for fast-dividing cells. However, therapeutic success cannot solely be accounted for by direct cytotoxicity. The eradication of cancer cells following DNA damaging therapeutics can further be attributed to stimulation of an anticancer immune response linked in part to the pattern recognition of cytoplasmic DNA [1,2,3,4]

### 1.1. Stimulator of Interferon Genes (STING) Pathway and Cancer 

STING, aka transmembrane protein 173 (TMEM173), connects cellular DNA damage with the immune system response. Under physiological conditions, DNA is compartmentalized in the nucleus and mitochondria. However, infection with DNA viruses, retroviruses or bacteria may result in the presence of cytosolic DNA, which acts as a danger signal and activates the immune response. Briefly, cytosolic DNA is detected in a sequence-independent manner by sensors such as cyclic GMP-AMP synthase (cGAS). cGAS converts ATP and GTP into 2′3′-cyclic GMP-AMP (cGAMP), which acts as a secondary messenger recognized by STING. STING, in turn, activates signaling pathways involving either tank-binding kinase 1 (TBK1)/interferon regulatory factor 3 (IRF3) or nuclear factor kappa B (NF-κB). These orchestrate the expression of interferons and other cytokines, leading to immune response activation and pathogen elimination [5]. 

### 1.2. Interferon Signaling 

Interferons are soluble cytokines, first discovered in 1957 as factors able to inhibit viral replication. They consist of three main types. Unlike type II (represented by a single IFN gamma syn. IFNG, produced solely by activated T lymphocytes), the type I interferon family consists of 17 cytokines, which are secreted and recognized by receptors on multiple types of cells. All type I interferons signal through the same dimeric receptor, though with different affinities, thus propagating slightly diverse transcriptional programs. Type III IFN, identified more recently in 2003, consists of three cytokines. They signal through a distinct receptor to type I IFNs. However, the interferon I and III type signaling converge, activating the same transcription factor complex (ISGF3), leading to the expression of a range of IFN regulatory factors (IRF) and IFN-stimulated genes (ISG) [6,7]. Transcriptional signatures of IFNs type I and III involve a similar set of genes, though activated to different levels [8]. Apart from interfering with viral infections, interferons exhibit proapoptotic and immunomodulating effects (reviewed by [9]). Interestingly, it has recently been shown that chemo- and radiotherapy may “mimic” viral infection through cytoplasmic DNA accumulation, which also induces interferon expression in a STING-dependent manner [10,11]. 

### 1.3. Cutaneous T Cell Lymphoma (CTCL) as a Research Model 

CTCLs are a heterogenous group of extranodal lymphomas, originating from mature T lymphocytes that persistently localize to the skin. The most common CTCL entities are mycosis fungoides (MF) and Sézary syndrome (SS), a leukemic variant of MF. Immune defects are a hallmark of CTCL pathogenesis, and the disease progression is related to an immunosuppressive cytokine profile (Th2) exhibited by malignant T cells. Defects in cytokine Th1 expression (such as interleukin (IL)-12 and IFN alpha), reduction of dendritic cell (DCs) activation and a decline in the number of CD8+ T cells may contribute to both impaired antitumoral and antimicrobial immunity [12].

### 1.4. Treatment Modalities in CTCL 

Therapeutic modalities are limited and aim for alleviating symptoms rather than being curative. Treatment is skin-directed or systemic depending on the disease stage [12,13]. Photochemotherapy is widely used in CTCL management and consists mainly of psoralens (e.g., 8–methoxypsoralen, 8–MOP) combined with UVA irradiation (PUVA). Skin-directed therapy with PUVA is preferentially used in patients at earlier stages with skin involvement alone or in combination with systemic therapies at later stages. Here, the photosensitizing agent (8–MOP) is taken orally prior to UVA exposure. Extracorporeal photopheresis (ECP) is a systemic treatment, recommended for patients in later stages, with generalized erythroderma and peripheral blood involvement. During ECP, patients are subjected to leukapheresis; collected white blood cells are treated with 8–MOP and UVA extracorporeally and subsequently reinfused [14,15]. Of note, formulations containing IFN alpha 2 (IFNA2) are routinely used in CTCL therapy and may be combined with other therapeutic modalities, such as PUVA or ECP. Allegedly, an IFN treatment ameliorates the above-mentioned immune defects, skewing towards a Th1 transcriptional profile [16].

Here, we demonstrate that the 8–MOP + UVA treatment activates the STING/cGAS pathway in CTCL cells. This promotes interferon expression, mainly of interferon lambda 1 (*IFNL1*), a representative of type III IFN. We also present an unbiased analysis of the transcriptional response to 8–MOP + UVA treatment, identifying 8–MOP + UVA-upregulated STING targets. Our results outline the close links between DNA damage and immune signaling in CTCL cells, which can open new avenues to advance therapeutic options in CTCL.

## 2. Materials and Methods

### 2.1. Cell Lines Used and Experimental Conditions 

The following cell lines were used in this study: MyLa2000 (derived from a plaque biopsy specimen from a patient with MF [17]), SeAx and Hut78 (derived from the peripheral blood of patients with Sezary syndrome [18,19]), and HaCaT (spontaneously immortalized human keratinocytes [20]). MyLa2000, SeAx and HaCaT were grown in GlutaMAX Dulbecco’s modified essential medium (DMEM), and Hut78 was grown in RPMI medium. Cell media were supplemented with 10% fetal bovine serum (FBS), 100-U/mL penicillin and 100-µg/mL streptomycin and RPMI additionally with 2-mM l glutamine (all from GIBCO BRL/Invitrogen, Auckland, New Zealand). 

### 2.2. Small Interfering RNA (siRNA) Transfection 

Transfection with specific siRNAs was performed for transient gene knockdown (see Supplementary Table S1 for the complete list of siRNAs). Hut78 and MyLa2000 were transfected by electroporation with Amaxa Nucleofector (Lonza, Basel, Switzerland) and Nucleofector Kit-T (Lonza), using the C-005 and A-030 protocols, respectively (0.5-nmol siRNA per 4 × 10^6^ cells). SeAx and HaCaT were transfected with Lipofectamine^®^ RNAiMAX (Invitrogen, Carlsbad, CA, USA). Briefly, 0.1-nmol siRNA and 5-µL Lipofectamine^®^ in 0.4-mL OPTI-MEM (GIBCO) were added to 10^6^ cells to the final volume of 2 mL for 4 h, before changing the medium. Knockdown efficiency was assessed by RT-qPCR 24 h after transfection. 

### 2.3. Photochemotherapy (8–MOP + UVA Treatment) 

CTCL cells were treated with 8–MOP + UVA 24 h after transfection or, in the case of experiments not involving transfection, after the addition of an equal volume of fresh medium. Cells were seeded at 0.5 × 10^6^/2 mL (transfected) or 10^6^/2 mL (untransfected) into 6-well plates, immediately after seeding treated with the indicated amounts of 8–MOP (1 or 2 µM; Fluka, St. Louis, MO, USA) for two hours before irradiation with UVA at 0.8, 1.6 or 2.4 J/cm^2^. HaCaT cells were seeded at 0.2 × 10^6^ and allowed to attach overnight before being subjected to 8–MOP + UVA treatment. 

TBK1 inhibitor BX795 (Tocris, Abingdon, UK) and ataxia-telangiectasia and Rad3-related (ATR) kinase inhibitor AZD6738 (Selleckchem, Houston, TX, USA) were added immediately after UVA irradiation.

Total RNA for RT-qPCR was harvested 24 h after treatment for Hut78, SeAx and HaCaT and 48 h after treatment for MyLa2000. Viability was assessed 24 h, 48 h and 72 h after treatment by propidium exclusion assay (see below). 

### 2.4. DNA-Damaging Agents 

In the experiments involving treatment with cisplatin (Accord Healthcare Ltd., North Harrow, UK) or etoposide (Sigma-Aldrich, St. Louis, MO, USA), cells were seeded at 10^6^/2 mL and treated with doses indicated in the figures. Total RNA isolation and viability assessment were performed as in the case of 8–MOP + UVA treatment. 

### 2.5. Viability Assessment 

Viability assessment was performed by propidium iodide exclusion assay. Briefly, cells were stained with propidium iodide (PI; 4 µg/mL; Sigma-Aldrich), incubated for 10 min and analyzed using a Cell Lab Quanta SC MPL flow cytometer (Beckman Coulter, Fullerton, CA, USA). The proportion of PI-negative (viable) cells was normalized to the vehicle-treated control (i.e., 0.4% *v*/*v* dimethyl sulfoxide, DMSO).

### 2.6. Real-Time PCR Analysis of Gene Expression 

Total cellular RNA was isolated using the NucleoSpin RNA kit (Macherey-Nagel, Düren, Germany). RNA concentration and purity were assessed using NanoDrop ND-1000, (Thermo Fischer Scientific, Wilmington, DE). RNA was transcribed into cDNA using the AffinityScript QPCR cDNA Synthesis Kit and oligo(dT) primers according to the manufacturer’s protocol (Agilent Technologies, Santa Clara, CA, USA). Real-time measurement of mRNA levels was performed with Stratagene 3005P qPCR System (Agilent Technologies) using TaqMan^®^ Gene Expression Assays (Applied Biosystems, Foster City, CA, USA) specific for each gene of interest (GOI; see Supplementary Table S1 for the list of the assays), apart from *STING* and *CGAS*, which were detected by SsoAdvanced Universal SYBR Green Supermix (Bio-Rad Laboratories, Hercules, CA, USA) with specific KiCqStart^®^ SYBR^®^ Green Primers (Merck, Darmstadt, Germany) (see Supplementary Table S1). Glyceraldehyde 3-phosphate dehydrogenase (*GADPH*) expression was used as a normalization control, and results were expressed as 2^(−ΔCt)^, where ΔCt = Ct GOI − Ct GAPDH. 

### 2.7. Micronuclei Detection 

Hut78 cells were seeded at 10^6^/2 mL; treated with 1-µM 8–MOP + 1.6 J/cm^2^ UVA and incubated for 24 h, 48 h and 72 h. Then, ca. 50,000 cells resuspended in phosphate buffered saline (PBS) were placed on a microscopic slide, air-dried and fixed in 4% PBS-buffered formaldehyde for 10 min. Nuclear and peri-nuclear DNA were stained using 1-µg/mL 4′, 6-diamidino-2-phenylindole, dihydrochloride (DAPI; Sigma-Aldrich) solution in water. Images were acquired using an ORCA R2 digital camera and HCImage software (both from Hamamatsu Photonics K.K., Hamamatsu, Japan). Experiment was performed in triplicate. Intact and micronucleated cells were counted manually in five random areas of each slide, and the percentage of micronucleated cells was calculated versus the total cell number. 

### 2.8. RNA-Seq Library Preparation and Sequencing 

Total RNA was isolated as described above. RNA quality and integrity were measured using the 2100 Bioanalyzer System (RNA 6000 Nano; Agilent Technologies), and only samples with an RNA integrity number (RIN) ≥ 8 were used. mRNA was enriched from 500-ng total RNA by RNA Purification beads (Illumina, San Diego, CA, USA), and a nondirectional RNA-Seq library was prepared using a TruSeq RNA Library Preparation Kit v2 (Illumina). Quality and concentration of the libraries were determined by a High-Sensitivity DNA kit (2100 Bioanalyzer; Agilent Technologies). Libraries were pooled, denatured and diluted to a final concentration of 1.8 pM. The pooled library was sequenced on the Illumina NextSeq500 (Illumina) with a single-end 75-bp sequencing kit.

### 2.9. RNA-Seq Data Analysis 

Quality control of sequence reads was done using the tools “FastQC” v0.11.2 [21], “RSeQC” v2.6.4 [22] and “fastq_screen” v0.11.4) [23].

Adaptors, low-quality bases and the first 12 bases and reads shorter than 25 nt were removed with “Trimmomatic” v0.39 [24] using settings “LLUMINACLIP:”, TruSeq3-SE.fa”:2:30:10 and HEADCROP:12 LEADING:3 SLIDINGWINDOW:4:15 MINLEN:25”.

Reads were mapped using “STAR” v2.6c [25] against the human genome (hg19). Up to two mismatches were allowed during the mapping, and the minimum number of overlap bases to trigger mates merging and realignment was set to five. Otherwise, default settings were used. Duplicate reads were removed using the “MarkDuplicates” function from the “picard” v2.6.0 software [26].

The “featureCounts” function of the “Rsubread” R package v1.32.4 [27] was used to quantify reads in exons. The Refseq hg19 gene annotation was used to assign reads to genes.

The “edgeR” v3.24.3 software [28] was used to perform a differential expression analysis. For this purpose, first, a model was defined indicating the experimental conditions and the library preparation batch information. Library normalization factors were calculated using the “calcNormFactors” function with the “TMM” algorithm. Tag-wise dispersion was calculated using the “estimateDisp” function with “robust = TRUE”. A gene-wise generalized linear model was fit with “glmQLFit”. Finally, differential gene usage was assessed using “glmQLFTest”. Resulting *p*-values were corrected for multiple testing using the “Benjamini-Hochberg” method. 

Gene set enrichment analyses of all annotated genes were done with the ranked log_e_ fold changes using the “gseGO” function in the “clusterProfiler” R package [29]. Related functions were used to visualize the results together with the “DOSE” R package functions [30]. To summarize the large list of enriched gene ontology (GO) pathways, the “emaplot” was produced, and the collapsed GO pathways were classified manually. For the final dotplot, the GO pathway with the most significant *p*-value was kept.

### 2.10. Statistical Analysis 

Experiments were performed in at least three independent biological replicates, with the exact number of replicates stated at each figure. The degree of transcriptional response varied, often resulting in skewed data distribution. Therefore, as the first step, data distribution was tested for normality using the Shapiro-Wilk test, and either a paired *t*-test or paired Wilcoxon test were performed for normally and non-normally (skewed) distributed data, respectively. A Shapiro-Wilk-threshold of 0.05 was set to distinguish the distribution. Statistical test applied and distribution type is stated in each figure legend (GraphPad Prism version 8.0.0 for Windows, GraphPad Software, San Diego, CA, USA).

## 3. Results

### 3.1. Photochemotherapy Induces Interferon Expression in CTCL Cells 

**Figure 1:**
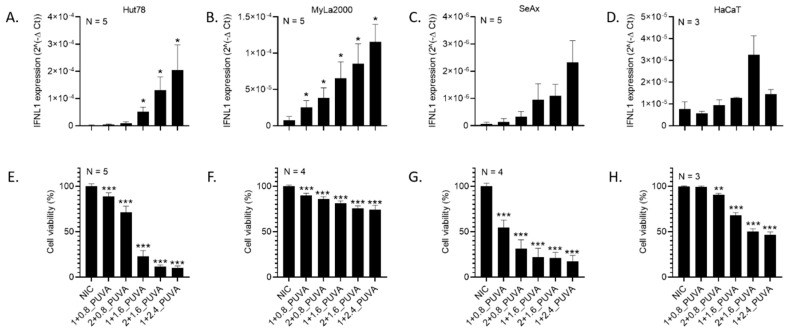
Cutaneous T-cell lymphoma (CTCL)-derived cells express interferon lambda 1 *(IFNL1)* in response to 8–methoxypsoralen and UVA light (8–MOP + UVA), and its expression is proportional to the cell death. Expression of *IFNL1* in (**A**) Hut78, (**B**) MyLa2000, (**C**) SeAx and (**D**) spontaneously immortalized human keratinocytes (HaCaT) treated with increasing doses of 8–MOP + UVA were measured by RT-qPCR and corrected for *GAPDH* expression. Viability of (**E**) Hut78, (**F**) MyLa2000, (**G**) SeAx and (**H**) HaCaT was evaluated by propidium iodide exclusion assay. Error bars represent ± SEM of the indicated N repeats. * *p* < 0.1, ** *p* < 0.05 and *** *p* < 0.01. NIC—not irradiated control and PUVA—8–MOP + UVA treatment; in the treatment description, the first number refers to the 8–MOP concentration in µM and the second to the UVA dose in J/cm^2^.

To test if the current CTCL therapeutic regime induces an interferon response, we treated three established CTCL-derived cell lines, namely Hut78, MyLa2000 and SeAx, with increasing doses of 8–methoxypsoralen (8–MOP) and UVA light (referred to as 8–MOP + UVA or PUVA). Additionally, HaCaT cells, immortalized human keratinocytes, were included as a positive control, since they were previously reported to express *IFNB1* in a STING-dependent manner [31]. Notably, the treatment increased interferon expression in all cell lines, though the expression profiles differed markedly (Table 1). Neither *IFNA1* nor *IFNA2* (often used in CTCL immunotherapy as an adjuvant [16]) were expressed by any of the CTCL cell lines, although a moderate *IFNA1* increase could be seen in HaCaT cells. The expression of *IFNB1*, frequently referred to in the context of STING signaling [11], was markedly induced in MyLa2000, SeAx and HaCaT but not Hut78. *IFNG*, the only representative of type II interferons, was only detected in MyLa2000 and SeAx (Table 1). Interestingly, all cell lines, though to various extents, expressed *IFNL1* (a type III interferon) in response to the treatment. *IFNL1* expression levels were proportional to the applied 8–MOP and UVA doses (Figure 1A–D), as well as to cell death induced by the 8–MOP + UVA treatment (Figure 1E–H).

### 3.2. Expression of IFNL1 Is Related to DNA Damage 

**Figure 2:**
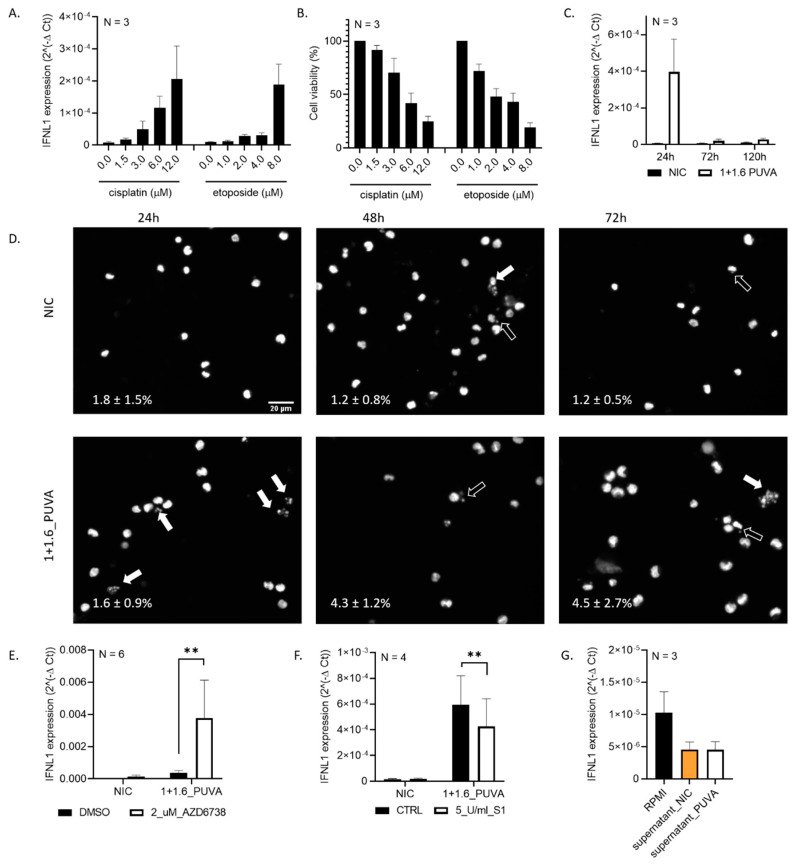
*IFNL1* expression in 8–MOP + UVA-treated Hut78 may result from acute DNA damage rather than micronuclei formation. (**A**) *IFNL1* expression upon treatment with commonly used genotoxic chemotherapeutics, cisplatin and etoposide. (**B**) Hut78 viability following treatment with cisplatin and etoposide. (**C**) *IFNL1* expression in Hut78 following 8–MOP + UVA treatment as a function of time. (**D**) DAPI staining of 8–MOP + UVA-treated Hut78 cells; solid white arrows indicate nuclei of cells undergoing apoptosis; empty arrows indicate micronuclei. Percent of micronucleated cells stated in the bottom-left corner in each photo. (**E**) *IFNL1* expression in cells treated with 8–MOP + UVA and ataxia-telangiectasia and Rad3-related (ATR) kinase inhibitor AZD6738; distribution: skewed, test: paired Wilcoxon. (**F**) *IFNL1* expression in cells incubated with S1 nuclease, specific for single-stranded DNA (ssDNA) and treated with 8–MOP + UVA; distribution: normal, test: paired *t*-test. (**G**) *IFNL1* expression in Hut78 cells treated with fresh RPMI medium, with supernatant collected from untreated and with supernatant collected from 8–MOP + UVA-treated cells; test: paired *t*-test. Error bars represent ± SEM of the indicated N repeats. ** *p* < 0.05.

Due to its planar structure, 8–MOP intercalates into DNA and, upon photoactivation by UVA, binds covalently to pyrimidine bases. This can cause replication roadblocks, leading to replication fork stalling, DNA breaks and apoptosis [32]. 8–MOP + UVA treatment has been reported to induce DNA damage detected by markers such as phosphorylated histone H2AX and phosphorylated checkpoint proteins (CHK1 and CHK2), substantiating that its cytotoxicity is effectuated via DNA damage [33,34]. Interferon expression (albeit, primarily type I interferon) has previously been related to DNA damage and the presence of free cytosolic DNA [35,36]. We were intrigued by the *IFNL1* increase in response to 8–MOP + UVA. Therefore, we asked if this interferon is induced by other types of genotoxic stress. Indeed, cisplatin and etoposide upregulated *IFNL1* in a dose-dependent manner (Figure 2A,B and Supplementary Figure S1). Analysis of the *IFNL1* expression as a function of time showed that, in Hut78 cells, *IFNL1* expression peaked around 24 h after 8–MOP + UVA treatment and then decreased, almost reaching basal levels after 72 h (Figure 2C). Previously, the activation of inflammatory signaling at three–five days following the genetic insult was reported [10,11] and ascribed rather to micronuclei formation than an immediate response to DNA damage. Micronuclei result from perturbed mitosis when cells with unrepaired or aberrantly repaired DNA breaks progress through mitosis. In our experimental setting, we did not observe an increased formation of micronuclei at 24 h post-8–MOP + UVA, which would coincide with the peak of *IFNL1* expression (Figure 2D); therefore, we speculate that damaged DNA, rather than micronuclei-contained DNA, may trigger *IFNL1* expression. 

Activation of the STING pathway and interferon expression is also related directly to DNA damage and the presence of cytosolic DNA, especially single-stranded DNA (ssDNA). Moreover, the inhibition of enzymes involved in DNA damage signaling and repair such as ataxia-telangiectasia-mutated (ATM) augment interferon expression [36,37]. The latter may result from an increased content of cytosolic DNA, which serves as a danger signal sensed by cGAS. In-line with these findings, we also observed that impairing the DNA damage response with AZD6738, a chemical inhibitor of ataxia-telangiectasia-mutated and Rad3-related (ATR) kinase, used at a subtoxic concentration, further augmented *IFNL1* expression induced by 8–MOP + UVA (Figure 2E). Additionally, *IFNL1* expression levels were slightly reduced following incubation with the S1 nuclease, specific for ssDNA (Figure 2F). This finding further suggests a link between ssDNA and *IFN1* expression.

In our setting, *IFNL1* expression was proportional to the cell death rate (Figure 1 and Figure 2A,B). In order to determine if the source of *IFNL1* was the cells with heavily damaged DNA, massively undergoing apoptosis or a bystander effect in viable cells [38], we incubated Hut78 cells with fresh RPMI medium, with supernatant from untreated cells or with supernatant from cells treated with 8–MOP + UVA and collected 24h after irradiation. We did not observe the elevated *IFNL1* expression in Hut78 cells treated with conditioned medium, either from untreated or 8–MOP + UVA-treated cells. These results indicate that, most probably, the primary source of *IFNL1* are cells undergoing apoptosis rather than a bystander effect in surviving cells (Figure 2G).

Collectively, these findings indicate that the *IFNL1* expression induced by 8–MOP + UVA in Hut78 cells results from DNA damage and is potentially triggered by the presence of cytoplasmic DNA.

### 3.3. IFNL1 Activation Following DNA Damage Is STING-Dependent 

We demonstrated that DNA damage causes a pronounced upregulation of *IFNL1* in Hut78 and MyLa2000 cells and, to a lesser extent, in SeAx and HaCaT cells. Previously, it has been demonstrated that STING activation can induce interferon expression following DNA damage [10,36]. Therefore, we asked if the STING pathway links 8–MOP + UVA-mediated DNA damage with interferon expression in CTCL. 

**Figure 3:**
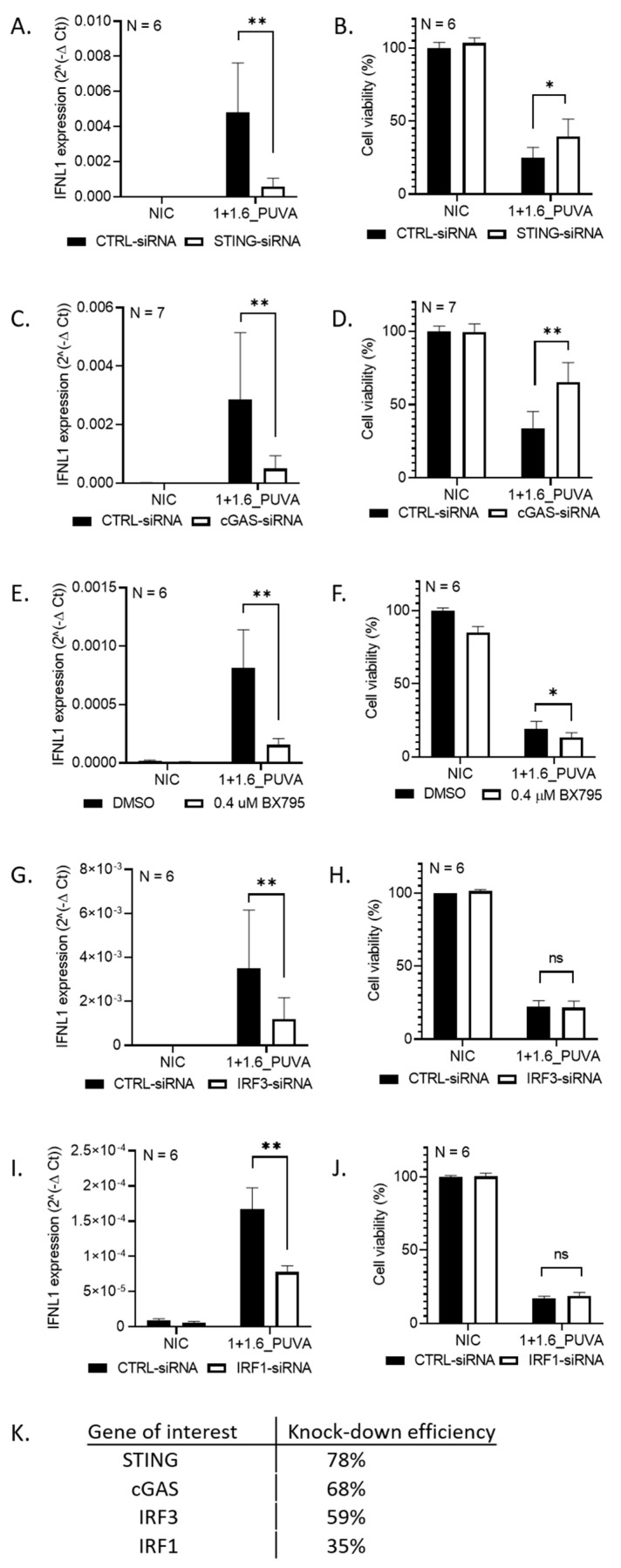
The Stimulator of Interferon Genes (STING) pathway is activated in Hut78 by 8–MOP + UVA treatment. Downregulation of alleged pathway elements by specific small interfering RNA (siRNA) or by a chemical inhibitor result in decreased *IFNL1* expression. Expression of *IFNL1* following 8–MOP + UVA treatment combined with (**A**) STING downregulation by siRNA, (**B**) cyclic GMP-AMP synthase (cGAS) downregulation by siRNA, (**C**) TBK1 inhibition by BX795 chemical inhibitor, (**D**) IRF3 downregulation by siRNA and (**E**) IRF1 downregulation by siRNA. Cell viability for respective treatments is presented in (**F**) for STING-siRNA, (**G**) for cGAS-siRNA, (**H**) for BX795-mediated TBK1 inhibition, (**I**) for IRF3-siRNA and (**J**) for IRF1-siRNA. (**K**) Transfection efficiencies for various siRNAs. Error bars represent ± SEM of the indicated N repeats. Statistics—normal distribution, paired *t*-test: (**A**–**E**) and skewed distribution, paired Wilcoxon: **(F–J)**. Choice of the statistical test was made based on the type of data distribution (see Methods). * *p* < 0.1, ** *p* < 0.05, ns–not significant.

Transient siRNA mediated knockdown of STING and cGAS abrogated *IFNL1* expression in Hut78 (Figure 3A,C) and in MyLa2000 (Supplementary Figure S2C). We observed that 8–MOP + UVA-induced *IFNB1* (in MyLa and SeAx) or *IFNG* (in SeAx) did not appear dependent on the cGAS-STING axis (Supplementary Figure S2). In-line with earlier reports, *IFNB1* in HaCaT remained under control of STING (Supplementary Figure S2H) [31]. Interestingly, STING and cGAS depletion promoted survival following 8–MOP + UVA treatment in Hut78 cells (Figure 3B,D) but not in MyLa2000, SeAx or HaCaT (Supplementary Figure S2).

Tank-binding kinase 1 (TBK1) and interferon regulatory factor 3 (IRF3) have been reported to operate downstream from STING [5]. Moreover, Chen et al. showed that STING-dependent IFNL1 production is also controlled by IRF1 in response to radiation [10]. To test if these factors are also active in our setting, we downregulated them with specific siRNAs (targeting TBK1, IRF3 and IRF1) or blocked with chemical inhibitors (TBK1), using *IFNL1* mRNA levels as a marker of downstream pathway functionality. 

The depletion of TBK1 did not significantly decrease *IFNL1*. Since the knockdown efficiency was only partial, we speculated that the residual kinase activity may be sufficient to ensure *IFNL1* expression (data not shown). Instead, we resorted to a chemical inhibitor BX795, which efficiently blocked *IFNL1* upregulation, suggesting a role of TBK1 in our cell model (Figure 3E). Furthermore, the depletion of IRF3 and IRF1 suppressed *IFNL1* upregulation following 8–MOP + UVA (Figure 3G,I). We observed no cytoprotective effect of TBK1, IRF3 or IRF1 blocking (Figure 3F,H,J). 

Altogether, the above findings indicate that the STING pathway operates in CTCL cells, and its activation by 8–MOP + UVA-mediated DNA damage results in the expression of type III interferon *IFNL1*.

### 3.4. 8–MOP + UVA Treatment Elicits Changes in the Gene Expression Profile Partly Dependent on the STING Pathway 

Our data suggested an 8–MOP + UVA-induced signaling response that involves the STING pathway. To uncover the major transcriptome changes, we performed a series of RNA-Seq experiments in cells depleted for STING and treated with 8–MOP + UVA. Twelve cDNA sequencing libraries were constructed, including controls, each in three replicates. For statistics and the quality assessment of RNA-Seq reads, see Supplementary Table S2. 

**Figure 4:**
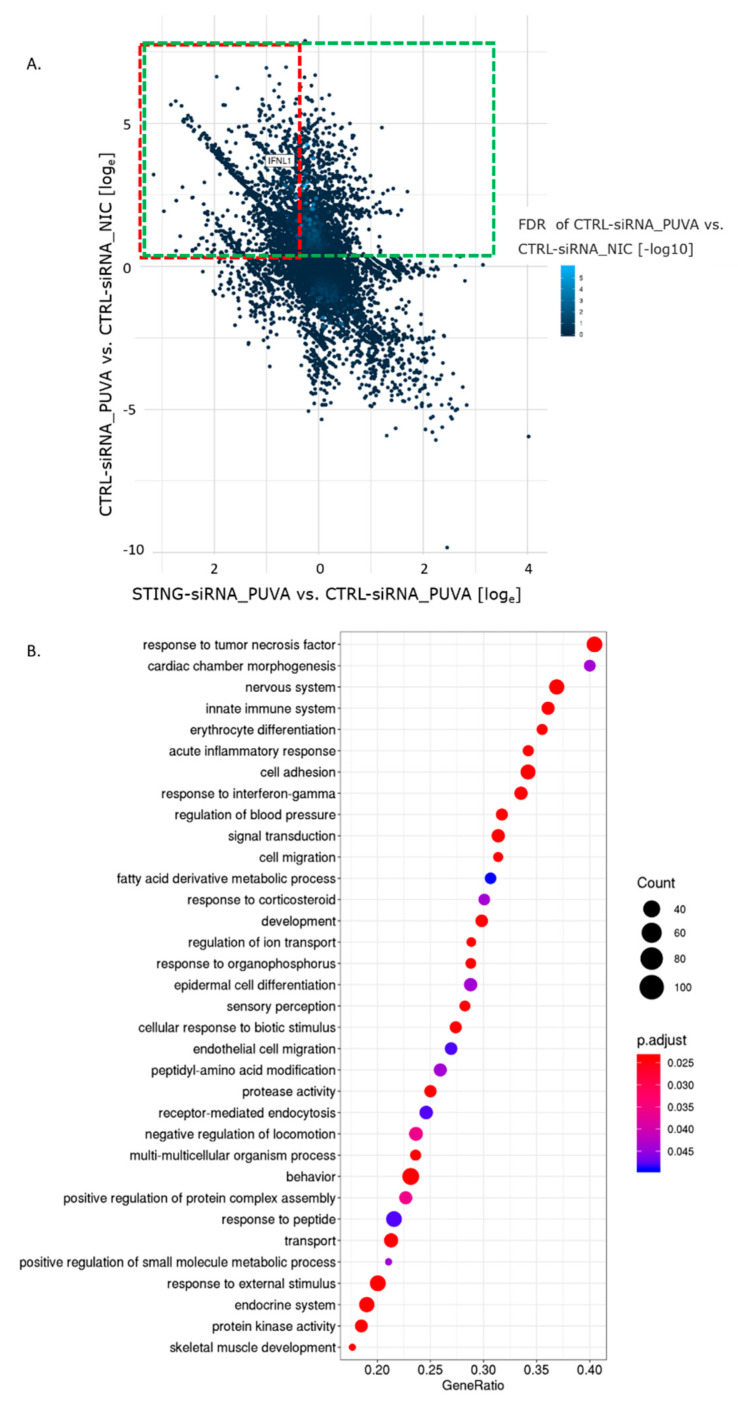
Unbiased analysis of the transcriptional response in Hut78 to 8–MOP + UVA treatment, performed by RNA-Seq. (**A**) 2D dot plot showing genes affected by 8–MOP + UVA treatment and STING downregulation. *X*-axis: gene expression ratio between STING-siRNA and CTRL-siRNA-transfected samples after 8–MOP + UVA (PUVA) treatment. *Y*-axis: gene expression ratio between NIC and 8–MOP + UVA samples, transfected with CTRL-siRNA; green frame: transcripts upregulated after 8–MOP + UVA treatment by at least 1 on the log e scale; red frame: transcripts upregulated by 8–MOP + UVA, which upregulation was suppressed by STING knockdown. Dot color scale: −log10 value of the false discovery rate (FDR) significance score of the differential expression (DE) analysis between the CTRL-siRNA_PUVA and CTRL–siRNA_NIC conditions. (**B**) Set of gene ontology (GO) pathways showing a significant change between NIC and 8–MOP + UVA (PUVA)-treated samples, transfected with CTRL-siRNA. They were sorted based on the gene ratio, which is the number of genes related to the GO term/total number of genes of interest. The complete list of pathways was summarized into broader categories shown in the *Y*-axis. The gene number ratio is shown in the *X*-axis, and adjusted *p*-values and counts are shown with a color scale and dot size.

Among the 21,133 annotated genes used in the analysis, the expression levels of 3316 were induced upon 8–MOP + UVA treatment (Figure 4A, upper-half of the *Y*-axis, green frame). STING knockdown negatively affected (suppressed) the upregulation of 80% of the transcripts (Figure 4A, top-left quadrant, red frame). 

### 3.5. Pathways Deregulated by 8–MOP + UVA Treatment 

The gene set enrichment analysis identified a number of pathways affected by 8–MOP + UVA treatment (Figure 4B and Supplementary Figure S3). The complete list of gene ontology (GO) biological processes was summarized into broader categories for better visualization (Figure 4B), whereas the detailed list of the enriched GO processes can be found in the Supplementary Materials (NIC_vs_PUVA_GSEA_detailed.xlsx). Among, altogether, 32 gene sets enriched by 8–MOP + UVA, processes such as a response to the tumor necrosis factor, innate immune system or acute inflammatory response were identified (Figure 4B). 

### 3.6. Hit Verification by RT-qPCR 

Next, we identified gene expressions that were significantly elevated by 8–MOP + UVA in a STING-dependent manner. The 10 top hits were subsequently verified by RT-qPCR. Candidate genes for RT-qPCR validation were selected if the same effect (STING-dependent upregulation after 8–MOP + UVA) was observed consistently across all three biological replicates. Briefly, the strategy for the selection of candidate genes was pursued as follows: Out of 21,133 transcripts detected in total, we first identified 3153 upregulated in all three replicates after 8–MOP + UVA. The upregulation of 222 transcripts was STING-dependent, as its knockdown reduced the transcripts by at least 25% in each of the three replicates. Eventually, we deselected the transcripts with the lowest expression (where the mean signal after 8–MOP + UVA was below 0.4 Reads Per Kilobase Million; RPKM). Altogether, we identified 66 genes fulfilling the above-described criteria (Supplementary Figures S4–S6). Among the top 10 were *IFNL1* (ranking as no. three), which was already analyzed, and *VGF* (no. five). The latter is primarily associated with the nervous system and, therefore, was excluded [39]. Subsequently, we included *OSGIN1* and *EGR1*, which ranked as 11th and 12th respectively. Moreover, we included *IRF7*, which has been reported to be the main regulator of IFN signaling [40], but, also, in turn, upregulated by IFN [8]. It did not appear in our ranking, as in one of the repeats, the effect of STING depletion was below 25%. The genes selected for further verification, together with their biological roles, are gathered in Table 2. RNA-Seq results for individual repeats, demonstrating the tendencies across the replicates, are shown in Supplementary Figures S4 and S5.

**Figure 5:**
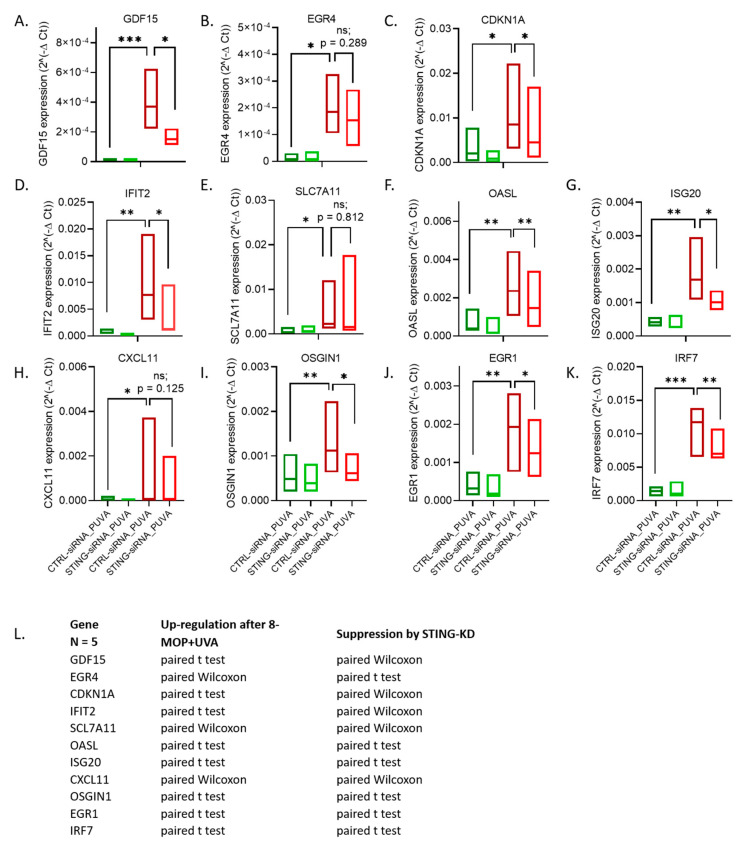
RT-qPCR verification of hits identified by RNA-Seq, i.e., transcripts upregulated by 8–MOP + UVA and remaining under STING control, largely confirms our findings. (**A**–**K**) Expression levels of each potential hit were measured in untreated (NIC) and subjected to 8–MOP + UVA (PUVA) treatment cells, transfected either with CTRL-siRNA or with STING-siRNA. (**L**) Statistical test used, chosen depending on the data distribution type (normal—paired *t*-test and skewed in at least one set—paired Wilcoxon test; see Methods). * *p* < 0.1, ** *p* < 0.05, *** *p* < 0.01, ns–not significant. STING knockdown efficiency: 82%.

All the potential hits selected for verification by RT-qPCR turned out to be upregulated by 8–MOP + UVA in Hut78 (Figure 5A–K). Of these, eight inductions (all apart from *EGR4*, *SCL7A11* and *CXCL11*) were STING-dependent (Figure 5). Meanwhile, most of the genes were upregulated in MyLa2000 following 8–MOP + UVA, with the exceptions of *ISG20* and *SCL7A11* (Supplementary Figure S7).

**Figure 6:**
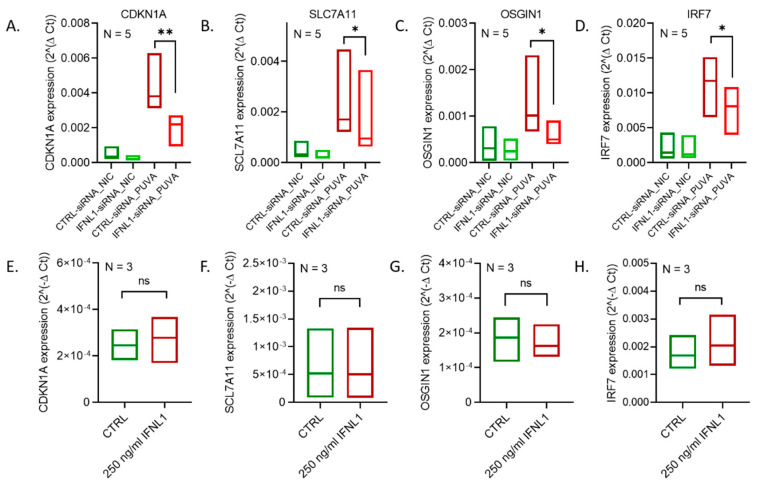
Expression of several of the potential hits remains under the control of IFNL1. Downregulation of IFNL1 abrogates the expression of *CDKN1A, OSGIN1, SCL7A11* and *IRF7* in response to 8–MOP + UVA. Hut78 cells were transfected with IFNL1-siRNA and, subsequently, treated with 8–MOP + UVA, and expression levels of the following genes were measured by RT-qPCR. (**A**) *CDKN1A*, (**B**) *SCL7A11*, (**C**) *OSGIN1* and (**D**) *IRF7*. Statistics: normal distribution, paired *t*-test: (**A**,**D**) and skewed distribution, paired Wilcoxon: (**B**,**C**). Choice of the statistical test was made based on the type of data distribution (see Methods). IFNL1 knockdown efficiency: 80%. Expression of (**E**) *CDKN1A*, (**F**) *SCL7A11*, (**G**) *OSGIN1* and (**H**) *IRF7* after treatment with soluble IFNL1 in the absence of DNA damage. Statistics: paired *t*-test. * *p* < 0.1, ** *p* < 0.05, ns – not significant.

In our research model, a set of genes was upregulated in response to 8–MOP + UVA in a STING-dependent manner. Attempting to further clarify the regulation mechanism, we tested whether IFNL1 depletion would affect their expression. Interestingly, we identified four genes (namely *CDKN1A, SCL7A11, OSGIN1* and *IRF7*) where 8–MOP + UVA induction was suppressed by IFNL1 depletion, suggesting that they remain under the control of IFNL1 (Figure 6A–D). This may suggest the induction downstream from IFN type III receptor for example via Janus kinase and signal transducer and activator of transcription (JAK/STAT) signaling [6]. We then assayed if the treatment with IFNL1 alone, in the absence of DNA damage, induced the target expression. However, it was not observed for *CDKN1A, SCL7A11, OSGIN1, IRF7* (Figure 6E,F) or any of the other gene candidates listed in Table 2 (data not shown). These findings suggest that the interaction with the IFN receptor alone, in the absence of stress stimuli (such as DNA damage), was insufficient for gene upregulation. 

## 4. Discussion

### 4.1. STING Pathway Activation in CTCL Cells 

In this study, we demonstrated that the currently used 8–MOP + UVA therapeutic DNA damage activates the STING/cGAS pathway in CTCL cells. This activation results in a transcriptional response involving a plethora of genes, with IFNL1 being a major induced cytokine. STING pathway activation, resulting in interferon expression, has previously been described both in malignant cells and in antigen-presenting cells of the tumor microenvironment. Sistigu et al. showed IFN type I secretion upon stimulation with anthracyclines by mouse fibrosarcoma cells [52]. Further, Corrales et al. demonstrated that STING agonists injected intratumorally induce the regression of established tumors, which was likely due to IFNB expression by immune, but not tumor, cells [53]. In both the above-mentioned studies, STING activation and interferon signaling contributed significantly to tumor eradication, regardless if tumor cells or immune cells were the source of interferons. In our experimental setting, malignant cells derived from the immune system (T-helper lymphocytes), which adds yet another layer of complexity. 

### 4.2. Immunomodulatory Effect of Photochemotherapy 

Originally, the efficiency of photochemotherapy was ascribed only to its cytotoxic effect. Lymphocytes (here, also, malignant T cells) appear to be more sensitive than monocytes, which implied partial selectivity of the 8–MOP + UVA treatment. However, during an ECP round, only 5–20% white blood cells are exposed to 8–MOP + UVA treatment. Still, the malignant clone seems to be specifically eliminated, with no general immunosuppression, suggesting an antigen-specific immunity. Tatsuno et al. demonstrated that 8–MOP + UVA induces immunogenic cell death (ICD) in melanoma cells, accompanied with danger signals, such as calreticulin exposure, ATP and HMGB1 release and, last but not least, IFNB1 secretion [54]. DNA-damaging agents have previously been reported to induce changes in dying cells, which would facilitate their recognition and killing by the immune system [55]. Briefly, it may involve attracting immune cells to danger signals released by dying cells and engulfing them by DCs, which, subsequently, present tumor antigens to selective cytotoxic T lymphocytes. These, in turn, expand and target patient’s CTCL cells [56]. We believe that the activation of the STING pathway, resulting in *IFNL1* expression by CTCL cells, is yet another link connecting DNA damage with immune system activation. However, it is not clear yet if the interferon expression following photochemotherapy may contribute to the alleviation of MF/SS symptoms. 

The use of a preclinical model in established cell lines allowed us to test and delineate the major pathway players after 8–MOP + UVA treatment. An exciting future step is to verify if circulating Sezary malignant cells recapitulate our findings. It would also be valid to establish if a correlation between interferon expression by circulating Sezary cells and clinical response to ECP exists. Moreover, it would be especially interesting to examine if the STING pathway is also activated upon PUVA treatment in mycosis fungoides patches and plaques. So far, UVA has been related to immunosuppression rather than immunostimulation, and no abscopal effect of PUVA has been demonstrated [57]. With further research, IFNL may become relevant in the therapy of CTCL in the hope of limiting side effects associated with the systemic use of IFNA2. Of note, IFN type III receptors are present on fewer tissues than type I, which may contribute to its more specific effect and better tolerability. Attempts to contain viral infections and to exploit their antitumor potentials have already been made [58].

Transcriptional response to 8–MOP + UVA. We report the STING-dependent upregulation of several genes following the 8–MOP + UVA treatment of CTCL cells. A subset (*IFIT2, OASL, ISG20, CXCL11* and *IRF7*; see Table 2) has previously been reported to be upregulated by interferons. The expression of four of the hits (namely, *CDKN1A, SCL7A11, OSGIN1* and *IRF7*) was abrogated by IFNL1 downregulation, which may suggest an auto- or paracrine expression manner. This would be in-line with a model of immune response suggested previously involving STING activation in response to genomic instability, which, in turn, leads to interferon secretion. Secreted interferon(s) bind, subsequently, to transmembrane receptors, eliciting autocrine and paracrine signaling and initiating the transcription of ISGs [59]. Moreover, the secretion of interferons has also been postulated to operate in feedback loops. For instance, knockout of the interferon IFN type III receptor subunit IL28R resulted in an impaired expression of *IFNL1* following ionizing radiation [10]. Similarly, IFNAR2-deficient sarcoma cells (Ifnar2−/−) failed to secrete IFN type I upon doxorubicin stimulation [52].

The mechanism of interferon signaling in response to 8–MOP + UVA, including the dependence of the STING transcriptional signature on IFN receptors and on the signaling cascade downstream from the receptors (JAK/STAT), should be carefully evaluated in CTCL cell lines and, if technically feasible, in patient-derived CTCL cells.

## 5. Summary and Conclusions 

Here, we uncovered that CTCL cells induce the expression of *IFNL1* in response to 8–MOP + UVA and that its expression is under the STING pathway control. Moreover, we showed an unbiased analysis of the transcriptional response to 8–MOP + UVA by means of RNA-Seq, which validated and further expanded the transcript response. We believe that our work supports the claim that photochemotherapy, apart from exerting a direct cytotoxicity, may have an immunomodulatory effect via STING pathway activation and interferon secretion. 

## Figures and Tables

**Table 1:** 8–Methoxypsoralen and UVA light (8–MOP + UVA) induces interferon (IFN) expressions in cutaneous T-cell lymphoma (CTCL) cell lines and spontaneously immortalized human keratinocytes (HaCaT).

	*IFNA1*	*IFNA2*	*IFNB1*	*IFNG*	*IFNL1*
Hut78	n.d.	n.d.	n.d.	(traces)	+++
MyLa2000	n.d.	n.d.	+	+++	+++
SeAx	n.d.	n.d.	+++	++	+++
HaCaT	+	n.d.	+++	n.d.	+

n.d.—interferon expression not detected. Increased IFN expression was marked with a plus (+) sign. (+)—mean IFN expression increased by a minimum of 1 cycle (ΔCt ≥ 1), (++)–ΔCt ≥ 2 and (+++)–ΔCt ≥ 3.

**Table 2:** Biological Roles of Genes Selected for RT-qPCR Validation.

Gene Candidate	Biological Role
*GDF15*	Growth/differentiation factor 15 (*GDF15*), belongs to the transforming growth factor β (TGF–β) superfamily; stress-induced, released in response to chemical or mechanical tissue injury [41]
*EGR4*	early growth response–4 (*EGR4*); zinc-finger transcription factor; a key regulator of T-cell differentiation and function; upregulated upon T-cell receptor (TCR) engagement, serves as a critical “brake” on T-cell activation [42]
*CDKN1A*	cyclin-dependent kinase inhibitor 1 (*CDKN1A*) regulates cell cycle progression by inhibiting cyclin dependent kinase 1 (CDK1) and CDK2 in e.g., response to genotoxic stress; facilitates cell entry into quiescence and senescence; acts to limit proliferation of stem cells; involved in the regulation of transcription, apoptosis, DNA repair, as well as cell motility [43]
*IFIT2*	IFN-induced gene with tetratricopeptide repeats 2 (*IFIT2*) syn. ISG54; one of cellular binding partners of STING; expressed in response to viral infections upon type I or III IFN; expression promotes apoptosis via a mitochondrial pathway [44]
*SLC7A11*	Solute Carrier Family 7 Member 11 (*SLC7A11*), syn. xCT; a subunit of an amino acid antiporter system, contributing to redox balance; its promoter contains sites recognized by STAT3 and STAT5 [45]
*OASL*	Oligoadenylate Synthetase-Like (*OASL*); IFN-induced; during infection with RNA viruses: binds to retinoic acid-inducible gene I (RIG–I) sensor sensitizing its activation and enhances antiviral signaling; during infection with DNA viruses: deactivates cGAS inhibiting IFN production [46]
*ISG20*	interferon-stimulated exonuclease gene 20 (*ISG20*); RNA exonuclease, induced by exposure to both type I (IFNA and IFNB) and type II (IFNG) IFNs; plays a role in mediating interferon’s antiviral activities [47]
*CXCL11*	C-X-C motif chemokine 11 (*CXCL11*) is a chemokine induced by IFNG and IFNB, and weakly by IFNA. One of three ligands of CXCR3, a receptor expressed i.a. on naïve T cells and upregulated by antigen presenting dendritic cells, resulting in Th1 polarization. CXCL11/CXCR3 interaction plays a role in immune cell activation, differentiation and migration, as well as in tumor suppression [48]
*OSGIN1*	Oxidative Stress Induced Growth Inhibitor 1 (*OSGIN1*) syn. OKL38 is a tumor suppressor, induced by DNA Damage in a p53-dependent Manner and regulated by histone Arg modifications [49]. It has been reported to be negatively regulated by xCT/SCL7A11 [50]
*EGR1*	early growth response–1 (*EGR1*) zinc-finger transcription factor; directly induces transcription of, i.e., TGFβ1, phosphatase and tensin homolog (PTEN), p53, fibronectin [51]
*IRF7*	Interferon regulatory factor 7 (*IRF7*); transcription factor essential for the induction of *IFN*–a/b genes [40]

## Supplementary Materials

The following are available online at https://www.mdpi.com/2073-4409/9/11/2452/s1: Supplementary Table S1. Full list of siRNAs used for gene knockdown and gene expression assays. Supplementary Table S2. RNA-Seq reads—data on quality and statistics. Supplementary Figure S1. *IFNL1* levels are upregulated in CTCL-derived cell lines following DNA damage. Supplementary Figure S2. STING pathway is activated in CTCL-derived cell lines following 8–MOP + UVA treatment. Supplementary Figure S3. Detailed gene set enrichment analysis. Supplementary Figure S4. RNA-Seq results for individual biological replicates (N = 3), demonstrating upregulation following 8–MOP + UVA across all the repeats. Supplementary Figure S5. RNA-Seq results for individual biological replicates (N = 3), demonstrating that STING knockdown suppresses 8–MOP + UVA-induced upregulation across all the repeats. Supplementary Figure S6. RNA-Seq analysis for the genes fulfilling the criteria described in the section “Hit verification by RT-qPCR” (STING-dependent genes upregulated after 8–MOP + UVA). Supplementary Figure S7. Majority of gene candidates upregulated following 8–MOP + UVA, identified by RNA-Seq in Hut78, are also affected in MyLa2000. NIC_vs_PUVA_GSEA_detailed.xlsx. File containing the detailed list of the enriched GO processes.
