# Supplementary material for: Photochemotherapy Induces Interferon Type III Expression via STING Pathway
Source: Cells. 2020 Nov 10;9(11):2452. doi: 10.3390/cells9112452 (PMC7697763; doi:10.3390/cells9112452)
Supplement: Supplementary file 1 [file cells-09-02452-s001.zip › Supplementary_materials_Biskup_et_al/Supplementary_materials_Biskup_et_al.docx]

**Supplementary tables:**

**Suppl. Table S1. Full list of siRNAs used for gene knock-down and Gene expression assays.**

| **Small interfering RNAs (siRNAs)** | |
| --- | --- |
| **Gene of interest** | **siRNA ID/Cat. no** |
| *STING* | 128591/AM16708 |
| *cGAS* | [129125](https://www.thermofisher.com/order/genome-database/details/sirna/129125?CID=&ICID=&subtype=)/AM16708 |
| *TBK1* | [134002](https://www.thermofisher.com/order/genome-database/details/sirna/134002?CID=&ICID=&subtype=)/AM16708 |
| *IRF3* | [106518](https://www.thermofisher.com/order/genome-database/details/sirna/106518?CID=&ICID=&subtype=)/AM16708 |
| *IRF1* | [106718](https://www.thermofisher.com/order/genome-database/details/sirna/106718?CID=&ICID=&subtype=)/AM16708 |
| *IFNL1* | [40532](https://www.thermofisher.com/order/genome-database/details/sirna/40532?CID=&ICID=&subtype=)/ AM16708 |
| **TaqMan gene expression assays** | |
| **Gene of interest** | **Assay ID/Cat. no** |
| *GAPDH* | Hs02758991_g1/4331182 |
| *IFNA1* | Hs03044218_g1/4331182 |
| *IFNA2* | [Hs00265051_s1](https://www.thermofisher.com/taqman-gene-expression/product/Hs00265051_s1?CID=&ICID=&subtype=)/4331182 |
| *IFNB1* | [Hs01077958_s1](https://www.thermofisher.com/taqman-gene-expression/product/Hs01077958_s1?CID=&ICID=&subtype=)/4331182 |
| *IFNG* | [Hs99999041_m1](https://www.thermofisher.com/taqman-gene-expression/product/Hs99999041_m1?CID=&ICID=&subtype=)/4331182 |
| *IFNL1* | Hs00601677_g1/4331182 |
| *TBK1* | [Hs00179410_m1](https://www.thermofisher.com/taqman-gene-expression/product/Hs00179410_m1?CID=&ICID=&subtype=)/4331182 |
| *IRF3* | [Hs01547283_m1](https://www.thermofisher.com/taqman-gene-expression/product/Hs01547283_m1?CID=&ICID=&subtype=)/4331182 |
| *IRF1* | [Hs00971965_m1](https://www.thermofisher.com/taqman-gene-expression/product/Hs00971965_m1?CID=&ICID=&subtype=)/4331182 |
| *GDF15* | Hs00171132_m1/4331182 |
| *EGR4* | Hs00231095_m1/4331182 |
| *CDKN1A* | Hs00355782_m1/4331182 |
| *IFIT2* | Hs00533665_m1/4331182 |
| *SLC7A11* | Hs00921938_m1/4331182 |
| *OASL* | Hs00984387_m1/4331182 |
| *ISG20* | Hs00158122_m1/4331182 |
| *CXCL11* | Hs00171138_m1/4331182 |
| *OSGIN1* | Hs00203539_m1/4331182 |
| *EGR1* | Hs00152928_m1/4331182 |
| *IRF7* | Hs01014809_g1/4331182 |
|  |  |
| Primers used with SsoAdvanced Universal SYBR Green Supermix | |
| Gene of interest | Assay ID |
| *GAPDH* | qHsaCED0038674 |
| *STING* | H_MB21D1_1 |
| *cGAS* | H_TMEM173_1 |

**Suppl. Table S2. RNA-Seq reads – data on quality and statistics.**

| **Sample Name** | **% Dups** | **% GC** | **Length** | **% Failed** | **Seqs** | **% Aligned** | **Aligned** |
| --- | --- | --- | --- | --- | --- | --- | --- |
| **CTRL-siRNA_NIC_rep1** | 39.9% | 49% | 75 bp | 9% | 7565336 | 80.3% | 6077779 |
| **CTRL-siRNA_NIC_rep2** | 58.1% | 48% | 75 bp | 18% | 25125516 | 83.9% | 21076519 |
| **CTRL-siRNA_NIC_rep3** | 49.0% | 46% | 75 bp | 18% | 20607920 | 83.5% | 17213586 |
| **STING-siRNA_NIC_rep1** | 43.4% | 50% | 75 bp | 9% | 9362276 | 79.4% | 7434264 |
| **STING-siRNA_NIC_rep2** | 57.4% | 48% | 76 bp | 18% | 55403771 | 84.7% | 46909952 |
| **STING-siRNA_NIC_rep3** | 59.1% | 48% | 75 bp | 18% | 15466407 | 82.6% | 12776192 |
| **CTRL-siRNA_PUVA_rep1** | 44.8% | 50% | 76 bp | 9% | 29688314 | 83.8% | 24887823 |
| **CTRL-siRNA_PUVA_rep2** | 68.5% | 48% | 75 bp | 18% | 27135038 | 84.8% | 23015261 |
| **CTRL-siRNA_PUVA_rep3** | 46.0% | 48% | 75 bp | 9% | 10667303 | 83.5% | 8902215 |
| **STING-siRNA_PUVA_rep1** | 63.4% | 47% | 75 bp | 18% | 58324886 | 84.5% | 49253585 |
| **STING-siRNA_PUVA_rep2** | 50.5% | 48% | 76 bp | 18% | 25953841 | 82.3% | 21353766 |
| **STING-siRNA_PUVA_rep3** | 64.4% | 48% | 75 bp | 18% | 18605342 | 82.2% | 15294320 |

**Supplementary figures:**

**Suppl. Fig. S1. *IFNL1* levels are up-regulated in CTCL-derived cell lines following DNA damage.** *IFNL1* expression levels in **(A)** – MyLa2000 treated with cisplatin, **(B)** – MyLa2000 treated with etoposide. Cell viability in **(C)** – SeAx treated with cisplatin, **(D)** – SeAx treated with etoposide. Error bars represent ± SEM of the indicated N repeats

**Suppl. Fig. S2. STING-pathway is activated in CTCL-derived cell lines following 8-MOP+UVA treatment.** **(A)** – *IFNB1*, **(B)** – *IFNG*, **(C)** – *IFNL1* – expression in MyLa2000 following 8-MOP+UVA; **(D)** – MyLa2000 viability following 8-MOP+UVA; **(E)** – IFNB1, **(F)** – IFNG expression in SeAx following 8-MOP+UVA; **(G)** – SeAx viability following 8-MOP+UVA; H - IFNB1 expression in HaCaT following 8-MOP+UVA; I – HaCaT viability following 8-MOP+UVA; J – transfection efficiencies for various siRNAs. Error bars represent ± SEM of the indicated N repeats. Statistics: paired t test, apart from H – Wilcoxon paired test. Choice of the statistical test was made basing on the type of data distribution (see Methods). ** p < 0.05, *** p < 0.01

**Suppl. Fig. S3**. **Detailed gene set enrichment analysis.** The complete set of GO biological processes showing a significant change between CTRL-siRNA_NIC_vs. CTRL-siRNA_PUVA has been depicted as a net. Adjusted p-values and counts are shown with a colour-scale and dot size.

**Suppl. Fig. S4.** **RNA-Seq results for individual biological replicates (N = 3), demonstrating up-regulation following 8-MOP+UVA across all the repeats.** **(A)** – *IFNL1*; **(B)** – *GDF15*; **(C)** – *EGR4;* **(D)** – *CDKN1A*; **(E)**– *IFIT2*; **(F)** – *SCL7A11*, **(G)** – *OASL*; **(H)** – *ISG20*; **(I)** – *CXCL11*; **(J)** – *OSGIN*; **(K)** – *EGR1*, **(L)** – *IRF7* expression in CTRL-siRNA transfected samples either untreated or 8-MOP+UVA treated was measured by RT-qPCR.

**Suppl. Fig. S5. RNA-Seq results for individual biological replicates (N = 3), demonstrating that STING knock-down suppresses 8-MOP+UVA-induced up-regulation across all the repeats**. **(A)** – *IFNL1*; **(B)** – *GDF15*; **(C)** – *EGR4;* **(D)** – *CDKN1A*; **(E)**– *IFIT2*; **(F)** – *SCL7A11*, **(G)** – *OASL*; **(H)** – *ISG20*; **(I)** – *CXCL11*; **(J)** – *OSGIN*; **(K)** – *EGR1*, **(L)** – *IRF7* expression in 8-MOP+UVA-treated samples, transfected either with CTRL-siRNA or STING-siRNA was measured by RT-qPCR.

**Suppl. Fig. S6. RNA-Seq analysis for the genes fulfilling criteria included in the Table 2.** Gene expression (RPKM) was normalized to the mean value of untreated controls (NIC, CTRL-siRNA) and sorted starting from the highest up-regulation following 8-MOP+UVA. N = 3

**Suppl. Fig. S7. Majority of gene candidates up-regulated following 8-MOP+UVA, identified by RNA-Seq in Hut78, are also affected in MyLa2000.** Expression of **(A)** – *GDF15*; **(B)** – *EGR4;* **(C)** – *CDKN1A*; **(D)**– *IFIT2*; **(E)** – *SCL7A11*, **(F)** – *OASL*; **(G)** – *ISG20*; **(H)** – *CXCL11*; **(I)** – *OSGIN*; **(J)** – *EGR1*, **(K)** – *IRF7* in untreated and 8-MOP+UVA-treated MyLa2000 cells. Paired t test; N = 4 * p < 0.1, ** p < 0.05, ns – not significant
