# Supplementary figures and images for: Photochemotherapy Induces Interferon Type III Expression via STING Pathway
Source: Cells. 2020 Nov 10;9(11):2452. doi: 10.3390/cells9112452 (PMC7697763; doi:10.3390/cells9112452)

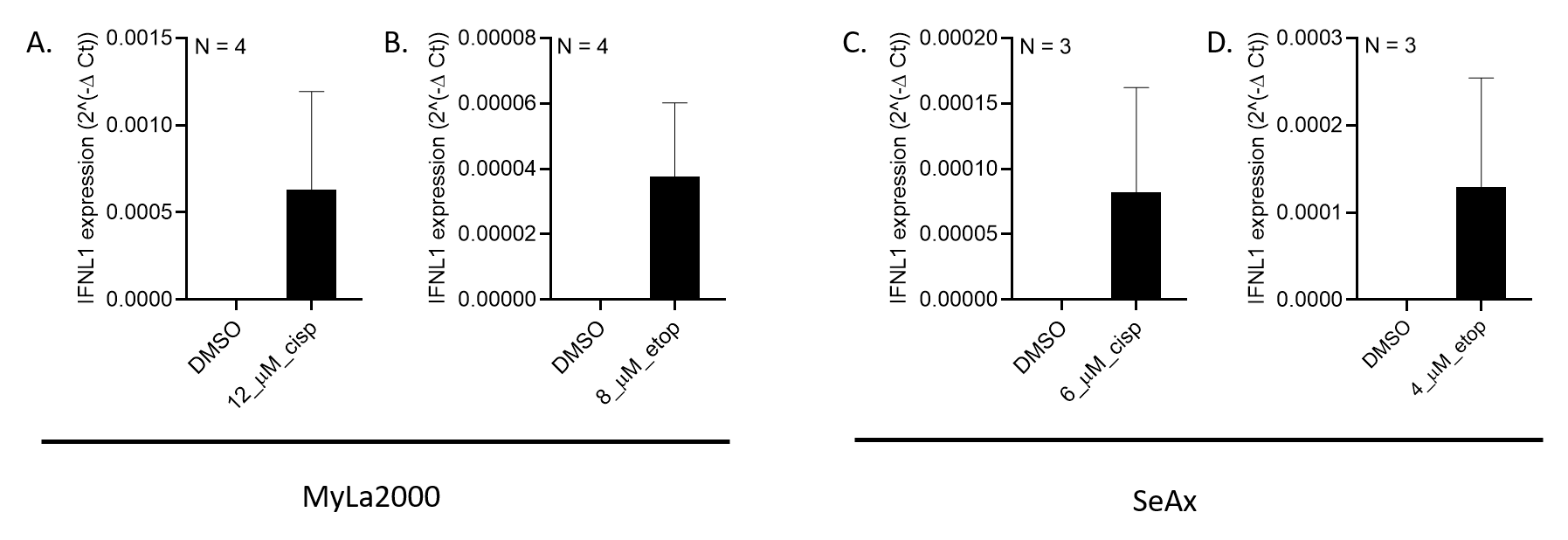

Supplement: Supplementary file 1 [file cells-09-02452-s001.zip › Supplementary_materials_Biskup_et_al/Suppl_fig_S1.tif]

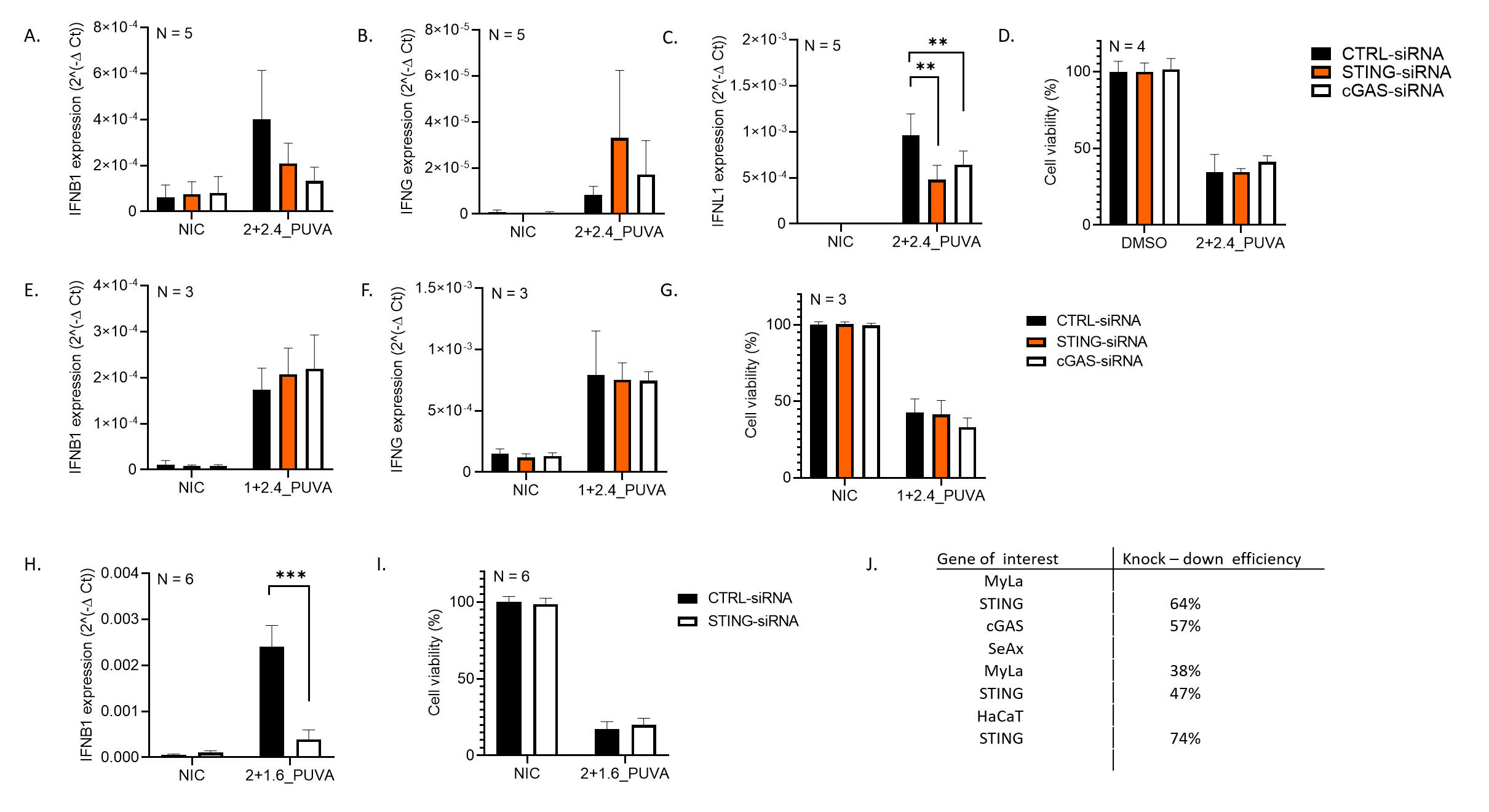

Supplement: Supplementary file 1 [file cells-09-02452-s001.zip › Supplementary_materials_Biskup_et_al/Suppl_fig_S2.tif]

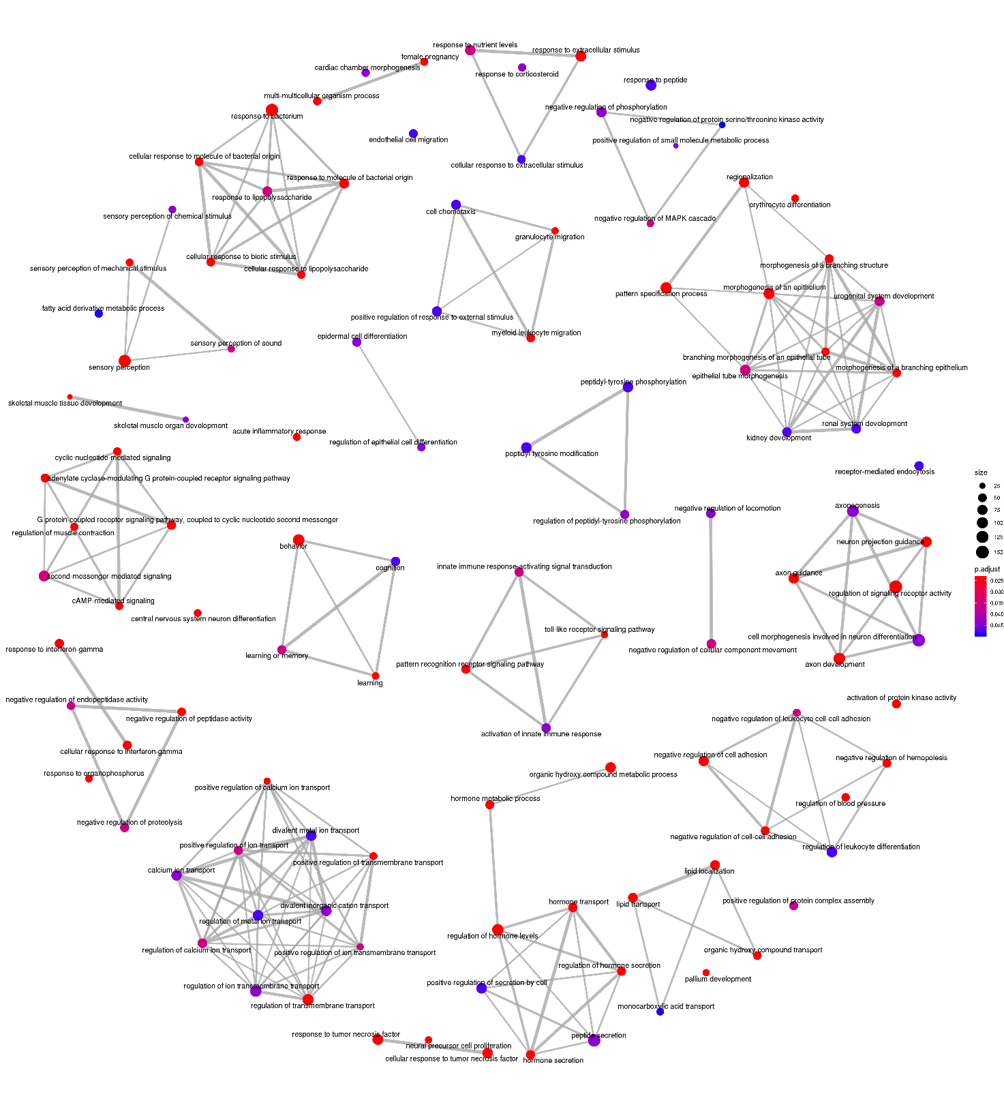

Supplement: Supplementary file 1 [file cells-09-02452-s001.zip › Supplementary_materials_Biskup_et_al/Suppl_fig_S3.tif]

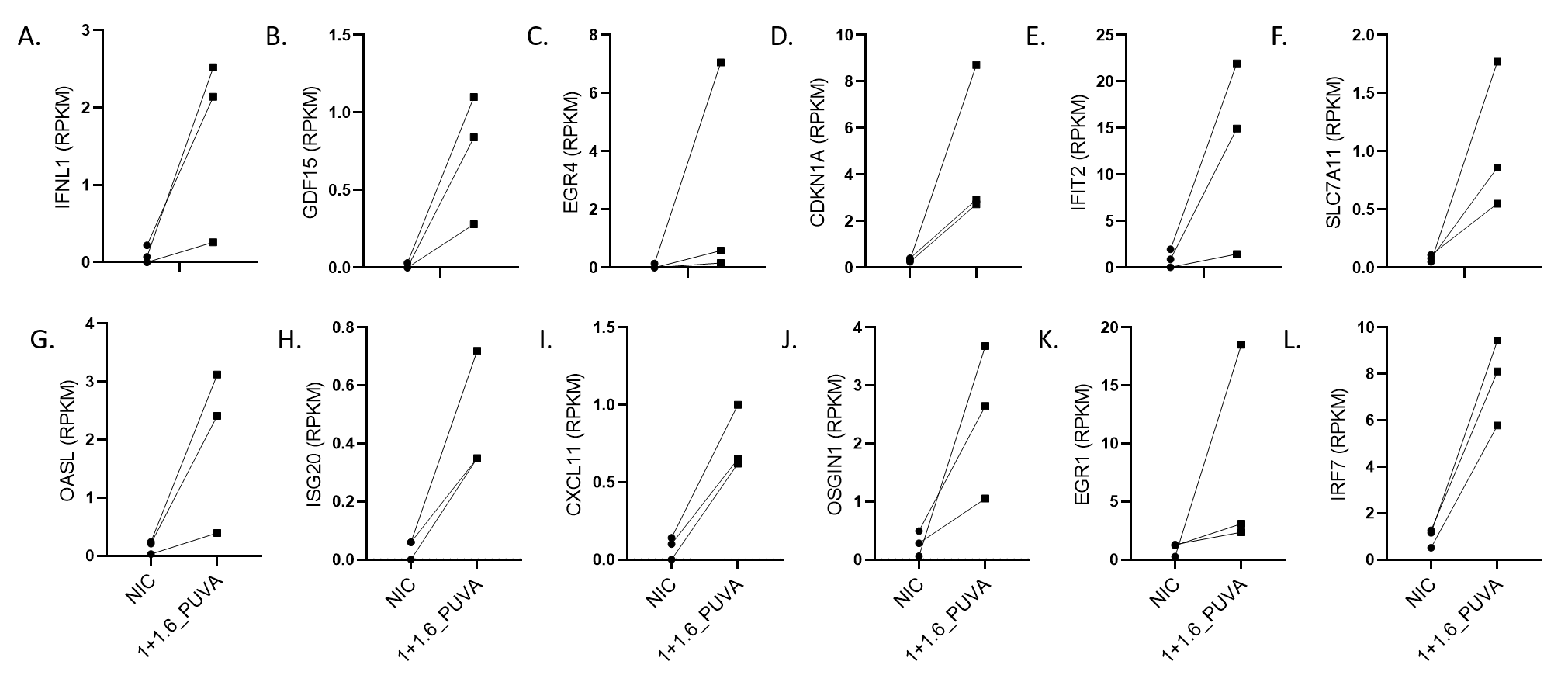

Supplement: Supplementary file 1 [file cells-09-02452-s001.zip › Supplementary_materials_Biskup_et_al/Suppl_fig_S4.tif]

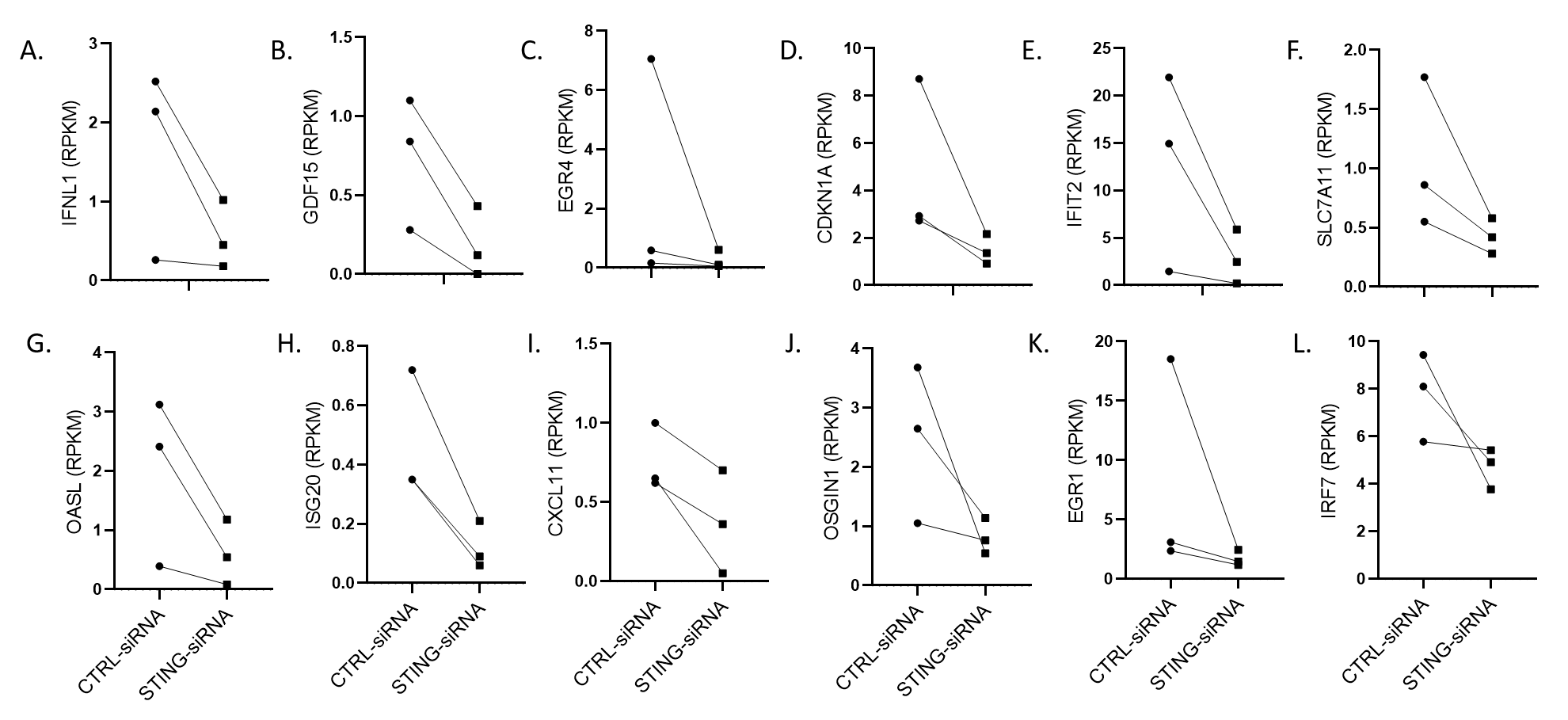

Supplement: Supplementary file 1 [file cells-09-02452-s001.zip › Supplementary_materials_Biskup_et_al/Suppl_fig_S5.tif]

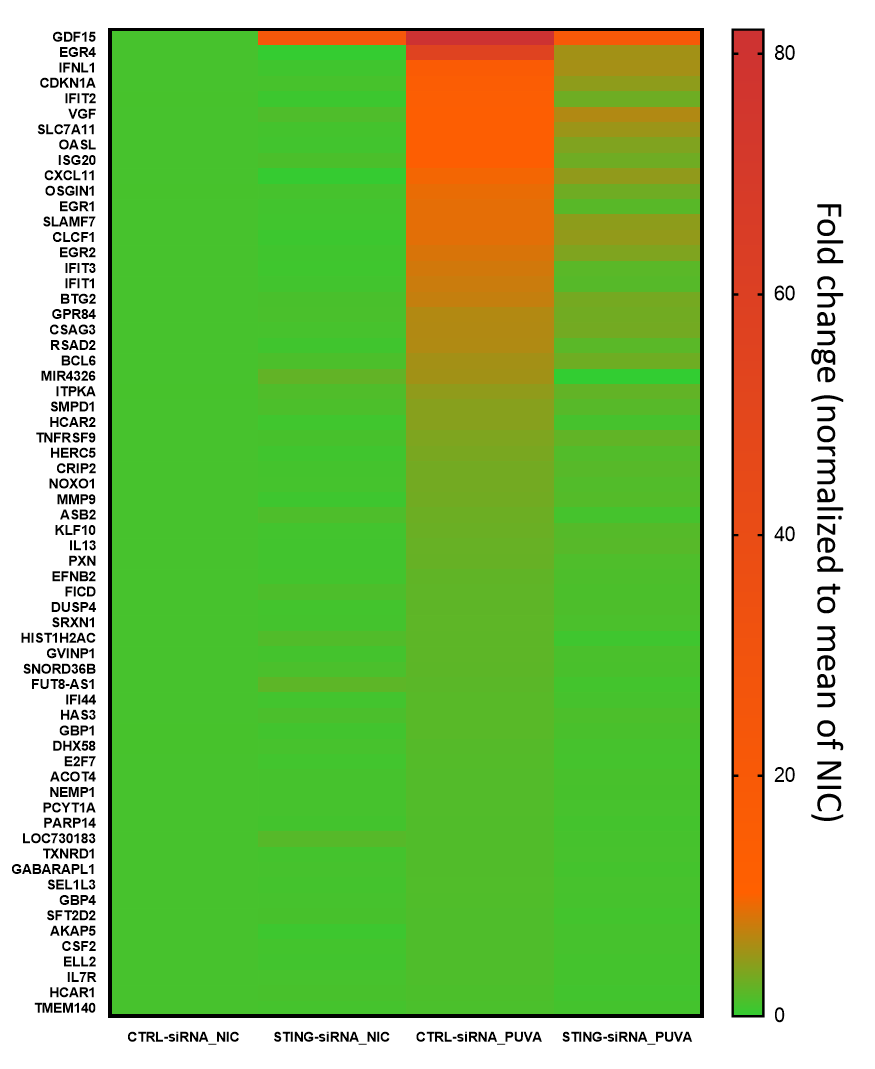

Supplement: Supplementary file 1 [file cells-09-02452-s001.zip › Supplementary_materials_Biskup_et_al/Suppl_fig_S6.tif]

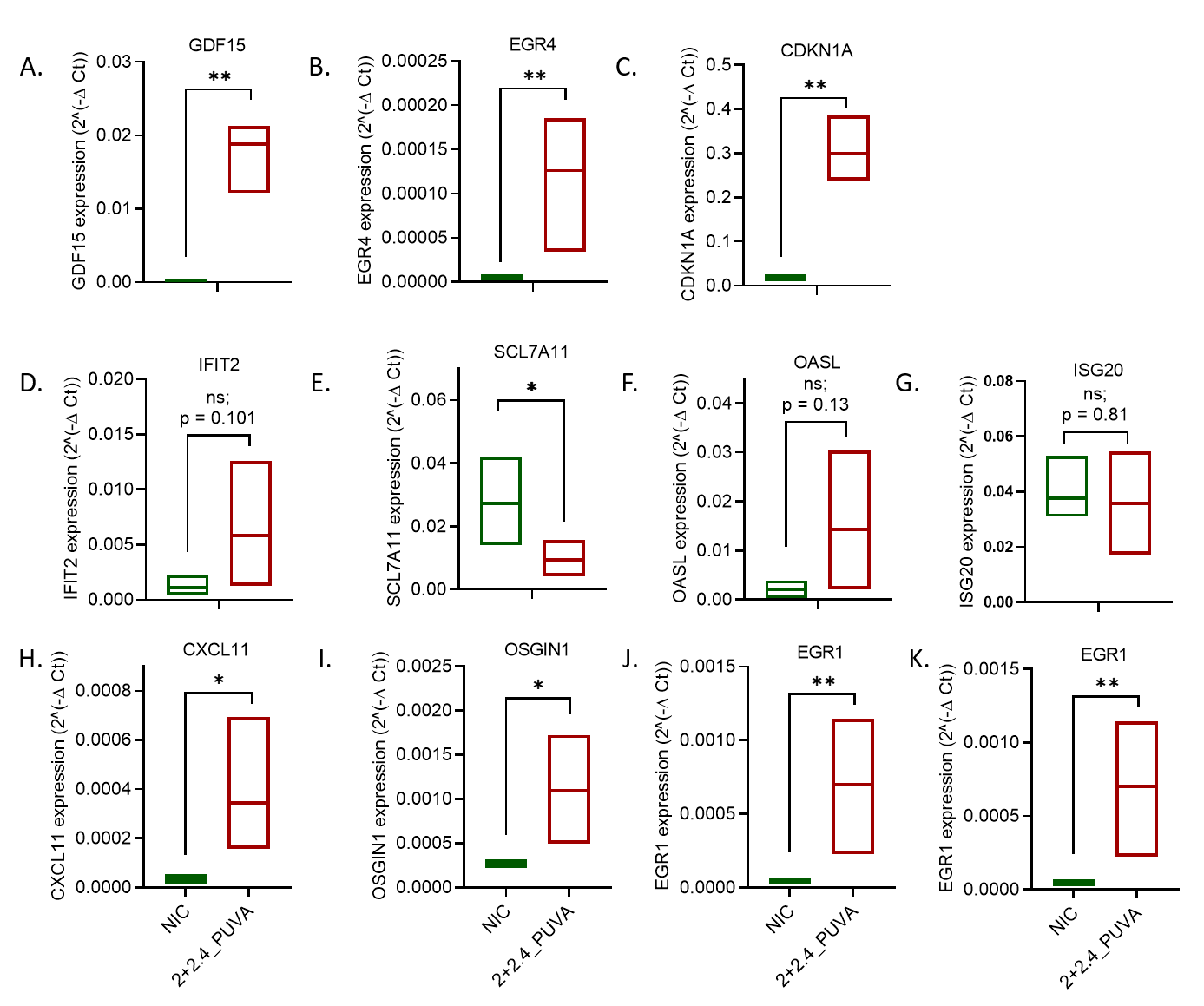

Supplement: Supplementary file 1 [file cells-09-02452-s001.zip › Supplementary_materials_Biskup_et_al/Suppl_fig_S7.tif]
